# Altered Perceptual Sensitivity to Kinematic Invariants in Parkinson's Disease

**DOI:** 10.1371/journal.pone.0030369

**Published:** 2012-02-17

**Authors:** Eran Dayan, Rivka Inzelberg, Tamar Flash

**Affiliations:** 1 Department of Computer Science and Applied Mathematics, The Weizmann Institute of Science, Rehovot, Israel; 2 Department of Neurobiology, The Weizmann Institute of Science, Rehovot, Israel; 3 Department of Neurology, The Sagol Neuroscience Center, Sheba Medical Center, Tel Hashomer and Tel Aviv University, Tel Aviv, Israel; University of Chicago, United States of America

## Abstract

Ample evidence exists for coupling between action and perception in neurologically healthy individuals, yet the precise nature of the internal representations shared between these domains remains unclear. One experimentally derived view is that the invariant properties and constraints characterizing movement generation are also manifested during motion perception. One prominent motor invariant is the “two-third power law,” describing the strong relation between the kinematics of motion and the geometrical features of the path followed by the hand during planar drawing movements. The two-thirds power law not only characterizes various movement generation tasks but also seems to constrain visual perception of motion. The present study aimed to assess whether motor invariants, such as the two thirds power law also constrain motion perception in patients with Parkinson's disease (PD). Patients with PD and age-matched controls were asked to observe the movement of a light spot rotating on an elliptical path and to modify its velocity until it appeared to move most uniformly. As in previous reports controls tended to choose those movements close to obeying the two-thirds power law as most uniform. Patients with PD displayed a more variable behavior, choosing on average, movements closer but not equal to a constant velocity. Our results thus demonstrate impairments in how the two-thirds power law constrains motion perception in patients with PD, where this relationship between velocity and curvature appears to be preserved but scaled down. Recent hypotheses on the role of the basal ganglia in motor timing may explain these irregularities. Alternatively, these impairments in perception of movement may reflect similar deficits in motor production.

## Introduction

There is increasing evidence for coupling between action and perception in humans and nonhuman primates [1]–[6]. Strong evidence derives from the discovery of the mirror neurons in the monkey ventral premotor [7] and inferior parietal cortices [8], which show close coupling between action production and action observation. The characteristics of the representations shared between perception and action are unclear [9]–[11]. Experimental evidence suggests that similar kinematic constraints and organizing principles, such as the “two-thirds power law” [12], underlie both a wide variety of movement generation tasks, as well as motion perception [13]–[19].

The two-thirds power law describes the strong relationship between the velocity of motion and the geometrical features of the path followed by the hand during planar drawing movements. For a variety of trajectories this relation can be described by:
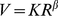
(1)where V is the tangential velocity at the end-point and R is the radius of curvature of the traced movement. K is the “velocity gain factor”, a parameter shown to be piecewise constant during entire movement segments [20]. For elliptical trajectories the exponent β in Equation 1 is very close to 1/3. Using an expression analogous to equation 1 with angular velocity (A) instead of tangential velocity and path curvature (C) instead of radius of curvature, the exponent β in the power law equation is close to 2/3. Thus, the velocity-geometry coupling captured by this mathematical formulation is often termed the “two-thirds power law”. While these formulations of the power law are used interchangeably in the literature, here for consistency we also use the term “two-thirds power law” when referring to Equation 1.

The two-thirds power law characterizes drawing movements [12], eye-movements [21], whole body movement during gait [22], and speech movements [23]. Interestingly, this motor invariant also constrains visual perception of motion [14]–[19]. In an influential study [14], subjects observing the movement of a light spot along an elliptical path were instructed to change its motion until it appeared to move most uniformly by controlling the movement's velocity-curvature relationships (i.e., the β exponent of the power law equation). Subjects tended to select as most uniform a motion corresponding closely to the two-thirds power law even though the spot's velocity could vary by up to 200% in this type of motion [15]. Compatibility with the two-thirds power law was also shown to clearly affect anticipation of perceived motion, both for handwriting movements [18] and for simple curvilinear trajectories [19]. These findings provide strong evidence that at the behavioral level similar constraints affect both generation and perception of movement.

Of note, both during motor production [12], [24] and visual motion perception [14], [15], the value of the exponent β even for ellipses is not strictly 1/3 but shows evident dependency on both the ellipse's eccentricity and movement duration, moving closer to 1/3 for faster motions and more eccentric ellipses [15].

A similar coupling was recently demonstrated using functional Magnetic Resonance Imaging (fMRI). In one study subjects viewed an abstract stimulus (a cloud of light spots) moving along elliptical paths either complying with or violating the two-thirds power law [17]. Motion complying with the power law resulted in selective activation in a widespread network of motor and motor-related brain areas, including the cerebellum and the basal ganglia. In another study human-like avatar animations were used as stimuli to test the effect of compatibility with motor invariants under relatively detailed and realistic visual settings. A network of regions in premotor cortex and the dorsolateral and dorsomedial prefrontal cortices showed preference to motion complying with biologically normal kinematics [25].

If action and perception are coupled, then a pathology in movement generation may be accompanied by a corresponding deficit in motion perception and possibly also in action recognition [26]. Yet, action recognition may be dissociated from higher-level motor processes in patients with brain damage [27], [28], suggesting that action recognition is not completely grounded in the motor system. Movement disorders, particularly Parkinson's disease (PD), a neurodegenerative disease resulting from the loss of nigrostriatal dopaminergic neurons, are useful for studying the coupling between action and perception. Deficits in motor control and sensorimotor integration in patients with PD have been extensively reported [29]–[34]. The motor performance of patients with PD does not fully show the kinematic regularities characterizing motor behavior of neurologically healthy subjects. For example, in point-to point reaching movements by healthy subjects the hand tends to follow a straight path with a bell-shaped velocity profile [35], [36], whereas in PD patients movements are nearly as straight as those of controls but lack their smoothness and symmetry [37]–[41]. Similarly, while curved hand movement paths of PD patients do not differ substantially from those of healthy controls, the velocity profiles show substantial abnormalities, lacking smoothness and including many small velocity peaks or displaying nearly constant movement velocity [39]–[41]. Unlike controls, patients also tend to pause at points of maximum curvature [40].

In addition to motor dysfunction, PD patients show a range of visuospatial dysfunctions [42]–[44], including deficits in motion perception in tasks requiring both lower and higher-level processing [42], [45]–[48]. Recent studies have also begun addressing the interplay between action and perception in PD. Patients with PD show less facilitation of simple motor responses through observation of similar actions than healthy controls [49], [50]. PD patients also show a weaker facilitation of motor signal transmission evoked by transcranial magnetic stimulation (TMS) than healthy controls, both while observing and imagining actions [51].

Here we examine whether the invariant features of movement generation, as captured by the two-thirds power law, also constrain motion perception in patients with PD as was shown in neurologically healthy volunteers. Another motivation for examining PD patients derives from our recent fMRI study showing that the basal ganglia in neurologically healthy humans respond preferentially to visual motion obeying the two-thirds power law [17], the basal ganglia being the major locus of dysfunction in PD. Utilizing a task used in previous studies with young healthy volunteers [14], [15], patients with PD and age-matched controls were asked to observe the rotation of a light spot along elliptical paths and to modify the velocity of the spot until it appeared to move most uniformly. The duration of the observed movements and the ellipse shapes were also manipulated [15].

## Materials and Methods

### Subjects

Twelve patients with idiopathic PD (9 women, 3 men; mean age 61.3±6.4 [SD]; mean years of education = 15.3±2.9), and 10 age-matched controls (5 women, 5 men; mean age 60.3±4.8; mean years of education = 14.9±2.1) participated in the study. Age and education differences between the two subject groups were not significant (t = 0.422 and t = 0.319 respectively). Background characteristics for the two subject groups are given in Table 1. Patients were recruited through the outpatient Movement Disorders Clinic at the Chaim Sheba Medical Center, Israel. All patients met the UK Brain Bank criteria for diagnosing idiopathic PD. Apart from one PD patient, all participants were right-handed. The patients were all tested during their “on” periods, while on their standard drug regimen. All participants gave their written informed consent. All procedures were conducted according to the principles expressed in the Declaration of Helsinki and were approved by the Ethics Committee of the Chaim Sheba Medical Center.

**Table 1:** Characteristics of PD patients and controls.

	PD (n = 12)	Controls (n = 10)
Age (yr)	61.3±6.4	60.3±4.8
Gender (M/F)	3/9	5/5
Education (yr)	15.3±2.9	14.9±2.1
MMSE	27.8±1.1	28.2±1.9
FAB	17.7±.5	17.8±.4
BDI	8.9±5.1	2.6±1.9

Mean values are displayed, along with standard deviations.

BDI, Beck Depression Inventory; FAB, Frontal Assessment Battery; MMSE, Mini-Mental State Examination.

#### Assessment of Parkinsonian symptoms, mood and cognitive function

PD patients were diagnosed at stages II and III of Hoehn and Yahr [52]. None had undergone surgical procedures for the treatment of PD. All patients were examined by a neurologist specializing in movement disorders (RI) using the motor subsection (part III) of the United Parkinson's Disease Rating Scale (UPDRS) [53]. This section's total scores range from 0–108; the sum of scores of 27 items for which 0 denotes no abnormality and 4 indicates full loss of motor function. None of the participants met the criteria for depression or dementia according to DSM IV. Furthermore, patients as well as controls were screened using the Beck Depression Inventory (BDI), Mini-Mental State Exam (MMSE) [54] and the Frontal Assessment Battery (FAB) [55]. Patients and controls did not differ in their MMSE scores (t = 0.49; not significant, ns) nor in their FAB scores (t = 0.10, ns). Scores for the BDI scale were significantly higher for PD patients (t = 3.17; p<0.01), yet none of the patients had a BDI score >19 which would indicate moderate to severe depression [56]. Table 2 displays the characteristics of the PD participants.

**Table 2:** Patient clinical characteristics.

	Sex	Age	PD Duration	H&Y Stage	Symptoms	Predominant side	Motor UPDRS	Treatment
1	F	62	3	2	B, R	L	15	LD,T
2	M	60	9	3	B, R, T	L	8	LD,DA
3	M	48	4	2	B, R, T	R	25	LD,DA
4	F	61	7	2	B, R, T	L	25	R,A,DA
5	F	58	5	3	B, R, P	R	22	S,DA
6	M	73	7	2	B, R, T	R	42	LD,A,S
7	F	57	6	2	B, R, T	R	19	R
8	F	57	7	3	B, R, T, P	L	23	LD,DA,R,T
9	F	63	10	3	B, R, T	L	16	A,R,DA
10	F	66	4	2	B, R, T	R	10	DA,R
11	F	69	3	2	B, R, T	L	8	A,S
12	F	62	4	2	B, R, T	L	40	S,T,DA

All patients, apart from patient 4 were right handed; H&Y, Hoehn & Yahr stage; Symptoms: T = tremor, B = bradykinesia, R = rigidity, P = Loss of postural reflexes. Treatment: A = Amantadine; S = Selegiline; T = Trihexyphenidyl; LD = L-dopa; DA = dopamine agonist.

### Tasks and Stimuli

The experiment was generated and maintained in real-time with the OpenGL Utility Toolkit (GLUT) over GNU C++ run on a Dell Latitude D505 laptop (screen resolution of 1400×1050 pixels) which also displayed the stimuli. The experiment was conducted in a quiet room. Participants were seated in front of the laptop screen and could choose their preferred viewing distance (typically ∼40 cm). Responses were collected via the laptop keyboard.

**Figure 1:**
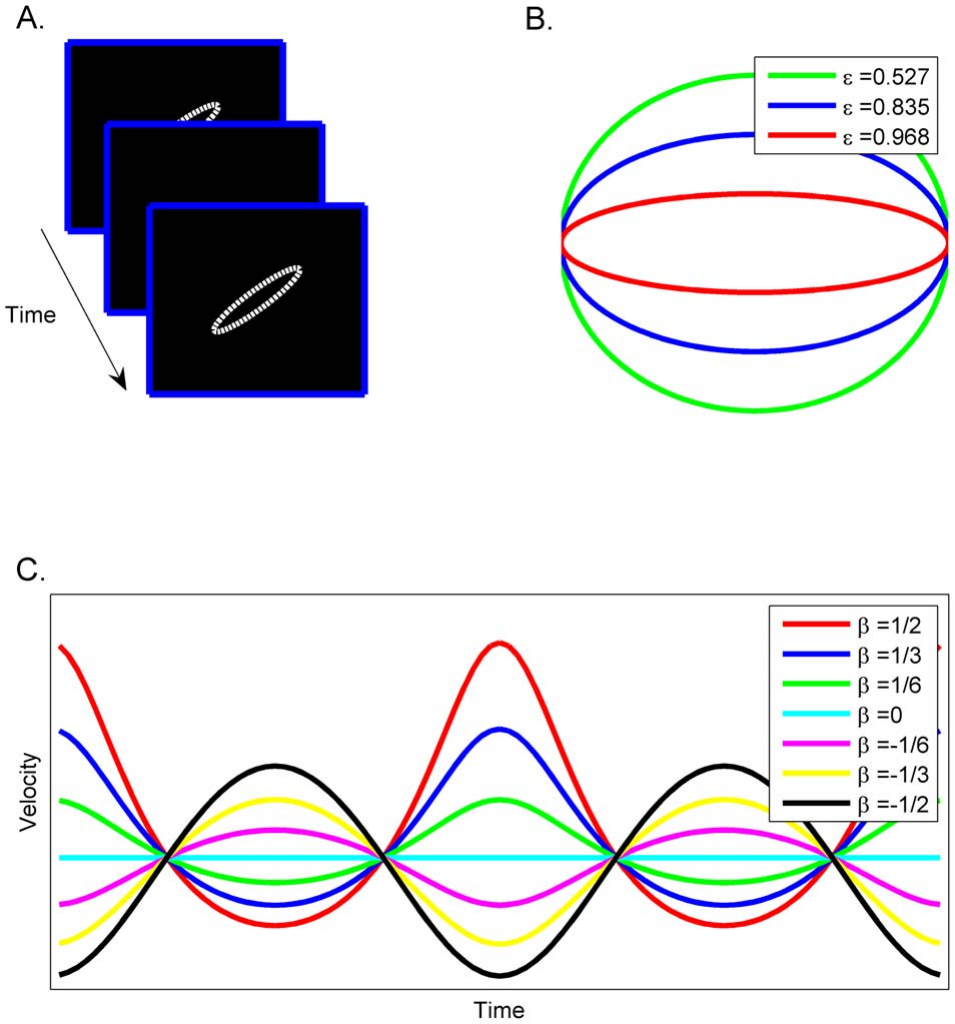
Experimental design and stimuli. (a) Subjects viewed a white light spot on the computer screen moving in elliptical trajectories. They were asked to modify its motion until it appeared to move most uniformly. (b) Ellipse eccentricity (ε) (c) Velocity profiles for the ellipse with medium eccentricity (ε = 0.835).

The experimental design and stimuli (Figure 1; Video S1) were similar to those used in previous studies [14], [15]. The stimulus was a white spot (approximately 0.6° of visual angle) on a dark background moving clockwise along elliptical paths (Figure 1A). Only the spot was visible during its movement. Subjects were asked to observe the motion of the spot and to modify its velocity until it appeared to move most uniformly, i.e., with the fewest changes in speed along the elliptical trajectory. They were informed that each trial had a unique solution.

The form of the elliptical trajectory and the duration of a complete cycle of the ellipse were manipulated during the experiment. Three elliptical shapes were created using three different eccentricities. The major semi-axis of the ellipses (BM) had a fixed length of 6.7 cm (visual angle of about 9.7°) and was rotated counterclockwise by 45°. The minor semi-axis (Bm) was 5.695, 3.885, or 1.675 cm giving a semi-axis ratio BM/Bm of 0.85, 0.55 and 0.25, corresponding to eccentricities of 0.527, 0.835 and 0.968, respectively (Figure 1B). The eccentricity (ε) of the elliptical path was defined as ε = (1-(B_m_/B_M_)^2^)^1/2^. The second manipulated factor was the tracing duration (T) of the moving spot, i.e., the time it took the spot to complete one cycle of the ellipse. Durations used were 1.5, 3.85 and 6.8 sec.

The paths of the light spot were pre-computed using MATLAB (Mathworks) and saved to a file which was read in real time by a custom-made computer program. The spot's initial speed, v_0_, was computed by inserting its initial curvature (C). Each time the scene was refreshed (approximately 150 times/s), the duration from the previous screen-refresh, Δt, was computed. The duration Δt, together with the speed of the previous scene-refresh, v_t_-Δt, enabled computation of the distance traveled along the path and, accordingly, the new position on the path. The curvature and speed of the next point were then calculated and this continuous routine was carried on until subjects changed the velocity-curvature relationship or terminated the trial.

In each experimental condition the instantaneous tangential velocity of the spot was related to the path's curvature through the power law equation. For consistency with our previous work [15], [17], we use the following formulation of the two-thirds power law: V = KR^β^, where V is the tangential velocity of the end-point, R is the radius of curvature and K is the velocity gain factor. The exponent β could take one of seven values: (−0.5, −0.333, −0.167, 0, 0.167, 0.333, 0.5). The 7 corresponding velocity profiles are displayed in Figure 1C for the ellipse with medium eccentricity. Velocity profiles for the most and least eccentric ellipses are displayed in Figure S1.

Since K was constant (see Introduction), the instantaneous tangential velocity was constant along the elliptical path only when the exponent β = 0. When β = 0.333, the movement complied with the two-thirds power law.

At the beginning of each trial the spot moved along the elliptical trajectory according to one of the different β values. These initial β values were counterbalanced across each session, so that each trial began with a different β value and the order of the β values within a session was randomized. The spot moved continuously along the elliptical path until the subject intervened by pressing either the left or right arrow keys or terminated the trial by pressing the spacebar. Pressing the laptop's arrow keys modified the spot's kinematics by either increasing or decreasing the value of the β exponent from its initial value. Subjects were instructed to use the two arrow keys, until the spot appeared to move most uniformly. There was no upper limit on the number of changes they could initiate and no instructions were given regarding reaction times (which were not considered as a variable). When the motion of the spot appeared to be most uniform, subjects were instructed to press the spacebar. At termination the final β exponent chosen by the subject was stored along with the whole history of the trial. A new trial began after 500 ms.

The experiment was divided into 3 sessions, one for each of the three tracing durations. Within each session, all three eccentricities were displayed (order was counterbalanced). Each session comprised 21 trials (7 initial β exponents * 3 eccentricities), giving a total of 63 trials in the entire experiment. At the beginning of the experiment, each subject was given a few practice trials, during which compliance with the task instructions was verified. All subjects took a short break between the experimental sessions.

### Data Analysis

The final β value chosen by subjects in each trial (β_f_) was stored and subjected to a repeated-measures Analysis of Variance (ANOVA), with the factors *tracing duration* and *eccentricity* serving as within-subject factors and *group* serving as between-subject factor. In all analyses Mauchly's test of sphericity was performed for all the repeated measures factors and, whenever this was found to be significant, Greenhouse-Geisser corrections were applied. Correlation analysis explored the relationship of the patients' background and clinical characteristics to their performance in the task. The analysis was based either on the Pearson product-moment correlation coefficient (Pearson's r), or on Spearman's rank correlation (r_s_) whenever one of the correlated variables was based on ranks. For all statistical tests, significance level was set at p<0.05.

## Results

Table 3 compares the mean and standard error (SE) of β_f_, the exponent chosen by subjects as producing the most uniform motion, for controls and PD patients. Both groups changed the β exponent a similar number of times before reaching a final decision (2.7±3.6 for controls, 2.65±3.5 for PD patients; t = 0.264, not significant, ns). For control subjects, movements with β_f_ >0 were chosen as the most uniform. Such movements tend to slow down during the more curved parts of the elliptical paths and to speed up during the straighter segments. The β_f_ values selected by the PD patients also differed from zero but were smaller than those selected by the control subjects. Hence, the motion chosen by PD patients as the most uniform was closer to movement at a constant Euclidean speed.

**Table 3:** Mean β_f_ choices along with the corresponding STEs across all the experimental conditions.

Condition	PD Patients	Controls
**T = 1.5**		
ε = 0.968	0.226±0.043	0.339±0.031
ε = 0.835	0.2±0.031	0.299±0.025
ε = 0.527	0.065±0.028	0.108±0.027
**T = 3.85**		
ε = 0.968	0.167±0.057	0.249±0.022
ε = 0.835	0.109±0.030	0.177±0.048
ε = 0.527	0.038±0.014	0.087±0.032
**T = 6.8**		
ε = 0.968	0.131±0.034	0.225±0.028
ε = 0.835	0.103±0.031	0.122±0.051
ε = 0.527	−0.065±0.034	0.016±0.017

Mean β_f_ values are presented for each of the tracing durations (T = 1.5, 3.85 and 6.8) and for each of the different eccentricities (ε = 0.527, 0.835 and 0.968).

**Figure 2:**
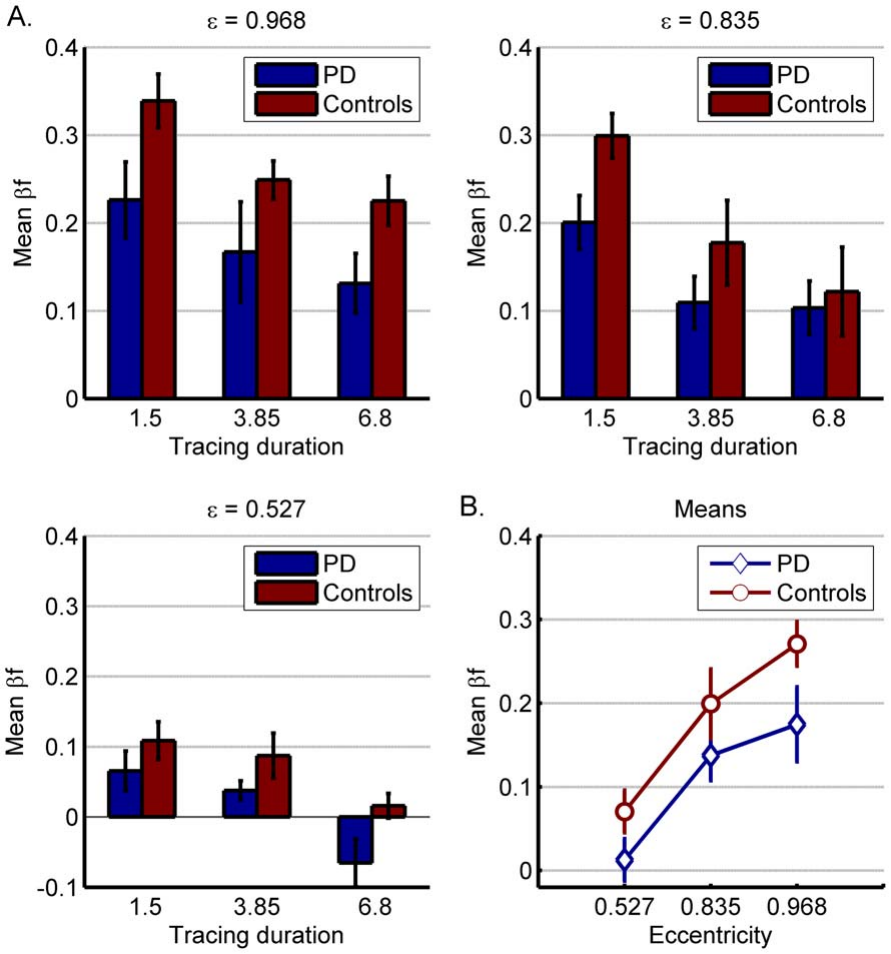
Mean β_f_ values chosen by PD patients and controls, for each of the three different eccentricities (A) and across the effect of tracing speed (B). Error bars denote SE.

A three-way ANOVA with eccentricity and tracing duration as within-subject factors and group as between-subject factor revealed no significant 3-way interaction among these three factors (F = 1.141; ns). We then continued to analyze the effect of each factor separately. First, we analyzed the effect of the shape (eccentricity) of the elliptical trajectory (Figure 2). A two-way repeated measures ANOVA with *eccentricity* as the within-subjects factor, and *group* as the between-subject factor revealed a main effect for eccentricity (F = 55.75; p<0.0001), whereby β_f_ values were larger (and closer to 1/3) with more eccentric elliptical trajectories. Across the 3 different elliptical trajectories (Figure 2A), PD patients chose smaller β_f_ values than the controls, as reflected by a significant main effect found for the *group* factor (F = 4.92; p<0.03). As can be seen in Figure 2, the differences between the β_f_ values chosen by patients and controls were smallest for the least eccentric ellipse (mean β_f_ = 0.013 PD patients; 0.071 controls) and largest for the most eccentric ellipse (mean β_f_ = 0.175 PD patients; 0.271 controls). However, on average, the difference between the β_f_ values selected by the PD patients and by the controls was maintained for all eccentricities (Figure 2B). Thus, overall the interaction between group and eccentricity was not significant (F = 0.71; ns), indicating that the differences between the two subject groups were stable across the three tested eccentricities.

**Figure 3:**
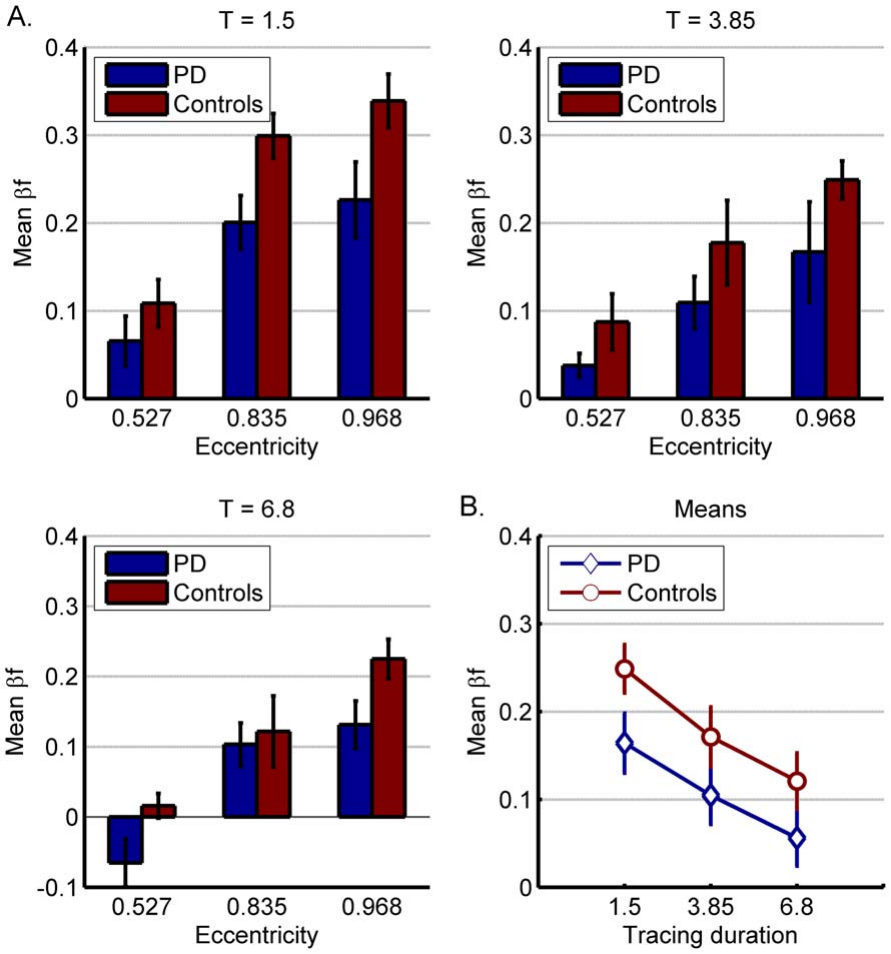
Mean β_f_ values chosen by PD patients and controls for each of the three tracing-durations (A) and across the effect of eccentricity (B). Error bars denote SE.

We next analyzed the effect of tracing duration on subjects' perceptual choices (Figure 3). The β_f_ values were subjected to a two-way repeated measures ANOVA with *tracing duration* as the within-subjects factor and *group* as the between-subjects factor. The β_f_ values were larger with shorter tracing durations (Figure 3A), resulting in a significant main effect for tracing durations (F = 43.31; p<0.0001). As shown in Figure 3A, mean β_f_ values chosen by the PD patients were consistently smaller than those chosen by the controls for each of the 3 tracing durations and the main effect for group was statistically significant (F = 4.92; p<0.03). For the shortest tracing duration, T = 1.5, the mean β_f_ value chosen by PD patients was 0.164 versus 0.249 for controls (Figure 3A). Similar differences were observed for both the medium and longest tracing duration (medium T = 3.85, β_f_ = 0.105 and 0.171; longest T = 6.8, β_f = _0.056 and 0.121 for PD patients and controls, respectively). As there were similar differences between patients and controls across the 3 tracing duration (Figure 3B), the interaction between *group* and *tracing duration* was not significant (F = 0.3; ns).

To examine whether patients' performance could be related to their background and clinical characteristics we first calculated a quantitative index for the difference between a patient's performance and that of the controls. The index was based on the Euclidean distance between the mean β_f_ value chosen by each patient and the mean β_f_ value chosen by all the controls. That is, for patient i, the difference index diff_i_ was calculated as diff_i_ = |β_fi_- β_fc_|, where β_fi_ is the patient's mean chosen β_f_ value and β_fc_ is the mean β_f_ value chosen by all the controls (both across all the experimental conditions).

The patients' background characteristics showed no correlation with the difference index (age, r = 0.069, ns; education, r = 0.037, ns). The difference index also showed no correlation with the patients' affective state (BDI scores, r_s_ = 0.070 - ns), nor with their cognitive state (MMSE scores, r_s_ = −0.026, ns). No correlation with the FAB test scores was calculated, as these scores were nearly maximal for all patients and comprised only two ranks. There was also no correlation between the difference index and the duration of the disease (r = −0.060, ns) nor with time since L-DOPA administration (r = 0.253, ns).

**Figure 4:**
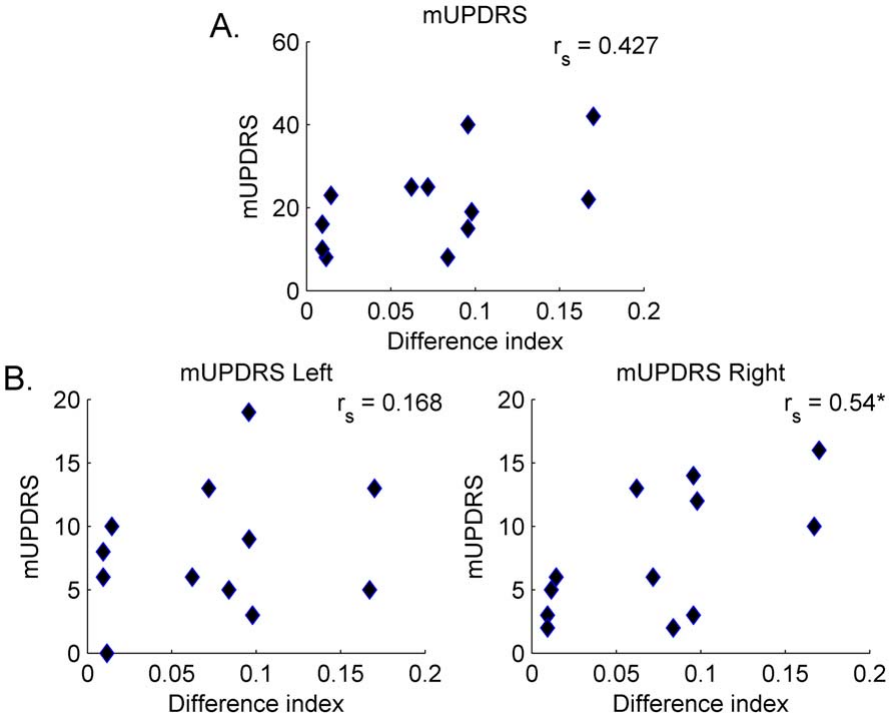
Scores of the difference index for each patient plotted against his/her total motor UPDRS score. (A) and against a composite scores for all left- and right-sided motor UPDRS items (B). * Statistically significant at p<0.05.

Correlating the difference index with the patients' motor UPDRS scores (Figure 4A) yielded stronger correlation coefficients. Patients' overall motor UPDRS scores were moderately correlated with the difference index (r_s_ = 0.427, ns). Composite scores for all right- and left-sided motor UPDRS items calculated for each patient revealed a moderate, statistically significant, correlation between the difference index and a composite score of all right-sided symptoms (r_s_ = 0.54; p = 0.033; Figure 4B). The correlation of the difference index and a composite score of all the left-sided symptoms yielded a much weaker correlation (r_s_ = 0.168, ns).

## Discussion

This study investigated whether the motor invariant, commonly referred to as the two-thirds power law, constrains motion perception in PD patients as it does in neurologically healthy individuals [14]–[19]. PD patients and age-matched controls were asked to modify the movement of a light spot until it appeared to move as uniformly as possible. Confirming earlier results [14], [15], neurologically healthy controls tended to choose movements obeying, or close to obeying, the two-thirds power law. This constraint was much less evident in the performance of patients with PD, who chose significantly smaller β_f_ values than age-matched healthy controls for all ellipses and tracing durations tested here.

Both the shape (eccentricity) of the elliptical path and the tracing duration of the moving light spot significantly affected subjects' β_f_ choices, affecting controls and patients similarly. As the differences between patients and controls were not restricted to a certain path nor a certain speed, they were probably not due to abnormalities in spatial perception nor to the geometry of the elliptical paths [57], [58]. Rather, these differences appear to represent a more global deficit in how visual motion is perceived in PD. Moreover, a similar effect of eccentricity and tracing duration in both subject groups rules out that the PD patients were merely guessing, since guesses should have resulted in similar β_f_ choices across all conditions.

For both patients and controls, as the ellipses became more eccentric, β_f_ choices were closer to 1/3 and further from 0, as found previously [14], [15], but to a lesser extent in PD patients than in controls. Both patients and controls chose larger β_f_ values closer to 1/3 for the fastest tracing duration and the value decreased as the tracing durations became slower, a finding also observed in young healthy subjects [15].

Interestingly, in movement production tasks the power law exponent (β) increases with movement speed [12]. The eccentricity of the movement path also influences the power law exponent. Under conditions where subjects were asked to generate drawing movements at their own comfortable speed, larger β exponents, which corresponded more closely with the two-thirds power law were obtained for movements with increasing eccentricity [24].

Both subject groups examined here were carefully matched for age, education and cognitive function, therefore these factors cannot account for the differences between patients and controls. Moreover, correlation analysis confirmed that age, education and cognitive function did not account for the intra-group differences within the patient group. One factor that we were unable to match among patients and controls was mood, as assessed with the Beck Depression Inventory (BDI). BDI scores for patients were significantly higher than those of the controls. However, as the patients' BDI scores were not correlated with their performance in the motion perception task, the contribution of mood to the current results seems unlikely.

One cause of differences between patients and controls may have been perceptuo-cognitive dysfunction, since a composite score of all right-side motor symptoms was positively and significantly correlated with the deviation from the average control β_f_ values. Right-sided motor deficits in PD are significantly correlated with state of general cognitive function [59], [60], as well as with more specific cognitive impairments of verbal memory, visuoperceptual skills and verbal fluency [60]. However, all the patients in the current study were non-demented and showed preserved cognitive and executive function, as assessed by DSM-IV criteria, the MMSE, and the FAB. Thus, any perceptuo-cognitive deficits underlying the differences observed here may reflect specific patterns of cognitive impairments, reminiscent of those observed in early stages of PD, often attributed to dysfunction of the cortico-basal ganglia-thalamo-cortical circuits [61].

Overall our results imply that the power-law relationship between velocity and curvature is preserved but is scaled down in PD. Yet, since the range of motion types subjects were able to choose from always obeyed a power-law relationship, the current results cannot rule out a more structured disruption in perception of motion. Our results may be related to the deficits in motion perception documented in PD [42], [45]–[48]. Such impairments may, at least in part, be due to retinal dysfunction due to dopaminergic depletion in the retina [42], [62], [63], yet cortical contributions cannot be ruled out [42], [45]. Patients with PD show deficits in the generation of both saccadic and smooth pursuit eye movements [64], [65]. However, as the power law in movement generation does not result from eye-movements [66], it is unlikely that our results derived from differences in eye-movement patterns [66]. Moreover, similar power law constraints in motion perception were reported when subjects were asked to fixate on a fixation spot while performing a perceptual decision task identical to that used here [15].

As the same invariants constrain movement generation and motion perception in healthy subjects [13]–[19], an appealing interpretation of our findings is that the performance of the PD patients in the perceptual task reflects similar deficiencies in the motor domain. Deviations from the kinematic regularities characterizing motor performance have been clearly documented in PD [32], [33], [37]–[41], [57]. Curved drawing movements of PD patients tend to be performed at an almost constant speed, showing multiple small velocity peaks or even a velocity plateau [39], [40]. The patients' curved velocity profiles were asymmetrical and were characterized by pauses at points of maximum curvature [40], clearly deviating from the smooth velocity profiles of motion complying with the two-thirds power law. However, the scribbling movements of patients with PD show co-variation between the velocity and curvature of the movement and tend to obey the two-thirds power law like those of healthy controls [57]. These movements were performed at the patients' velocity of choice, whereas here and in other previous reports [39], [40] all aspects of the visual trajectory of the moving dot were predetermined, thus making comparisons difficult. Moreover whether healthy subjects perceive motion as uniform largely depends on whether the visual path is constrained (ellipses) or unconstrained (as in scribbles) [14]. The perception of unconstrained movements may rely on different processes from those used for tracking a constrained path, a difference also postulated for execution of movement [57].

Requiring a precise representation of how velocity changes over time, perceiving and generating motion obeying the two-thirds power law necessitates accurate motor and perceptual timing. Extensive evidence suggests that fronto-basal ganglia networks are involved in the representation of time and timed behavior, with the basal ganglia appearing to play a central role [67]–[69]. PD patients show motor, sensory and perceptual timing deficits [70], so presumably the neuronal populations within the basal ganglia and related areas impaired in PD play an important role in the neural representation of time. Our findings may thus reflect deficiencies in the functioning of such timing mechanisms, suggesting that basal ganglia dysfunction affects time and velocity perception as well as the ability to control movement speed. In this context we note that PD patients show velocity estimation deficits [71], which are consistent with models of basal ganglia based timekeeping.

It was previously suggested that the two-thirds power law reflects motion at a constant equi-affine speed, which is the time derivative of the equi-affine arc-length, the latter being equivalent to Euclidean distance, weighted by the path curvature to the power of 1/3 [72]–[74]. Thus, the sensitivity to the two-thirds power law in motion perception as well as production suggests that internal motion representations may be based on equi-affine rather than on Euclidean velocities [17], [74], [75]. This hypothesis was more recently generalized to suggest that movement timing and duration may arise from a mixture of several geometries, particularly Euclidian, equi-affine and full affine geometries [74]. It was further speculated that many dynamically interconnected neuronal populations, most probably within different brain areas, may use different possible combinations of geometries which may influence movement timing. The known deficits of PD patients in motor timing [68] as well as the altered perceptual sensitivity to motion that follows the two-thirds power law, as observed in the current study, suggests that the neuronal populations within the basal ganglia and related areas which are impaired in PD play an important role in the neural representation of time. Thus, basal ganglia dysfunction may affect both time and velocity perception as well as the ability to control movement speed. This suggestion remains to be more fully explored in future experimental and theoretical studies.

## Supporting Information

Figure S1 **Velocity profiles used in the experiment.** (A). Velocity profiles for the most eccentric ellipse (ε = .968). (B) velocity profiles for the least eccentric ellipse (ε = 0.527).
(TIF)

Video S1 **Task and stimuli used in the experiment.** Shown are 3 consecutive trials for an ellipse with medium eccentricity (ε = 0.835) and medium tracing speed (3.85 sec).
(WMV)
