# Supplementary figures and images for: Altered Perceptual Sensitivity to Kinematic Invariants in Parkinson's Disease
Source: PLoS One. 2012 Feb 17;7(2):e30369. doi: 10.1371/journal.pone.0030369 (PMC3281839; doi:10.1371/journal.pone.0030369)

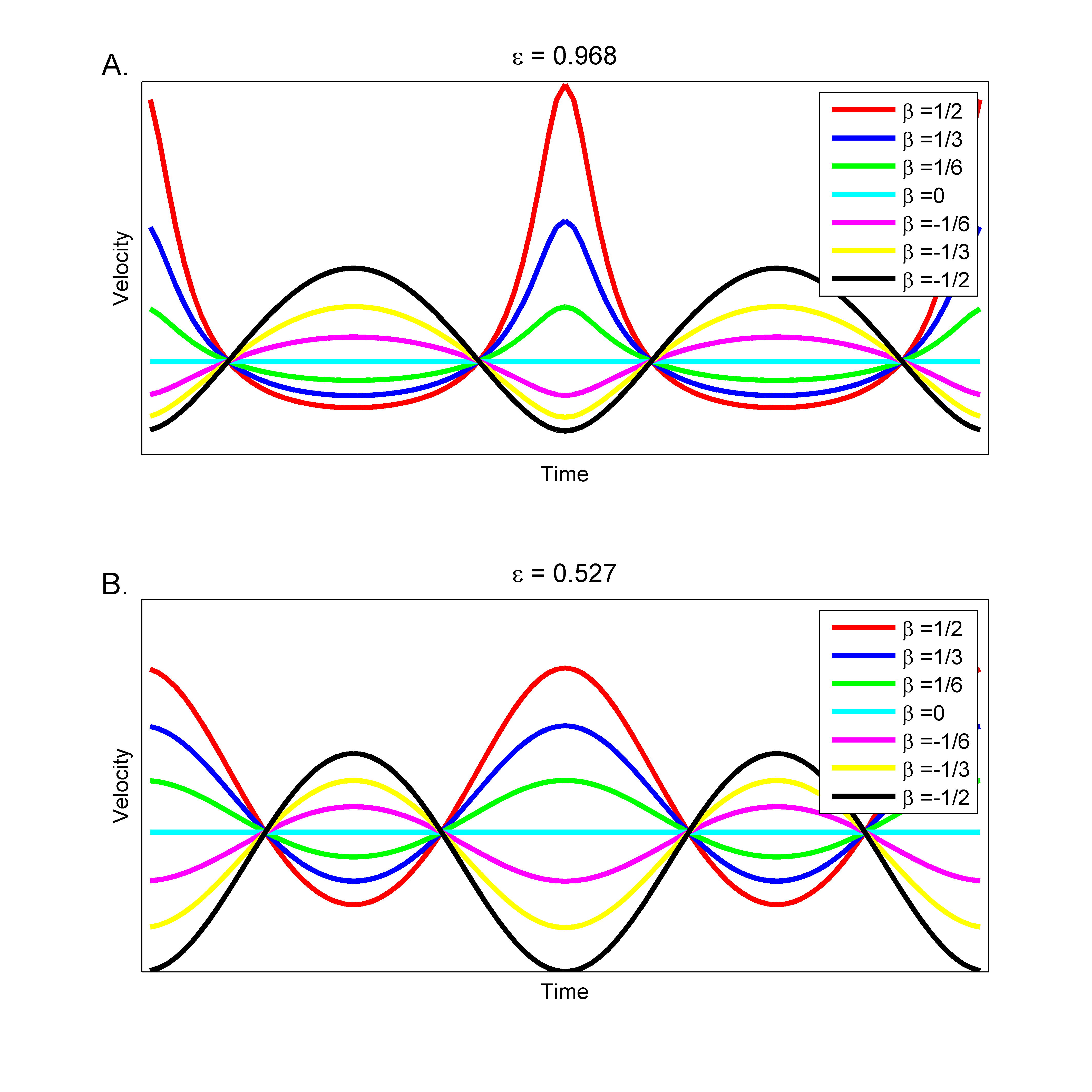

Supplement: Figure S1 — Velocity profiles used in the experiment. (A). Velocity profiles for the most eccentric ellipse (ε = .968). (B) velocity profiles for the least eccentric ellipse (ε = 0.527). (TIF) [file pone.0030369.s001.tif]
